# Advanced Haemodynamic Monitoring During Transfemoral Aortic Valve Replacement: A Prospective Pilot Study

**DOI:** 10.3390/life15111714

**Published:** 2025-11-05

**Authors:** Astrid Bergmann, Philip Woldt, Lena Steins, Nikolai Hulde, Janis Fliegenschmidt, Cornelia Piper, Tanja Rudolph, Vera von Dossow

**Affiliations:** 1 Heart- and Diabetes-Center Bad Oeynhausen, Ruhr-University Bochum, 44801 Bochum, Germanyvvondossow@hdz-nrw.de (V.v.D.); 2 Heart- and Diabetes-Center Bad Oeynhausen, Medical Center East Westphalia-Lippe, University Bielefeld, 33615 Bielefeld, Germany

**Keywords:** advanced haemodynamic monitoring, TAVI, pilot study, time-weighted average, intraoperative hypotension, intraoperative volume

## Abstract

This pilot study aims to compare advanced and standard haemodynamic monitoring during TAVI in terms of predicting and avoiding hypotension. Intraoperative hypotension influences postoperative outcomes by increasing mortality, renal failure, and cardiac complications. In TAVI (transaortic valve implantation), haemodynamic stability is essential because the patients are usually old and vulnerable. Fifty patients underwent transfemoral TAVI under standard anaesthetic care. Blood pressure was measured invasively, using Edwards Acumen sensors connected to a HemoSphere monitor. The signal was simultaneously fed to anaesthesia monitors. Patients were randomly divided into two groups: in the test group, the Edwards monitor with the HPI (hypotension prediction index) values was available to the anaesthetist, whereas in the control group, the HemoSphere monitor was covered. The primary endpoint of the study was the time-weighted average of intraoperative hypotension, which is calculated from the intensity and duration of hypotension, adjusted for the duration of surgery (TWA_65_). Secondary endpoints were the cumulative time of hypotensive episodes adjusted for the duration of the procedure (TWA_total_). No difference in intraoperative hypotension in terms of TWA_65_ between control and intervention group could be detected, the overall duration of intraoperative hypotension was reduced in the intervention group, and the administration of intraoperative volume was higher in the intervention group when compared to controls. The use of HPI during TAVI leads to improved haemodynamic stability, and this is particularly important in these extremely vulnerable patients. Not only is it possible to reduce overall intraoperative hypotension with HPI, but postoperative complications associated with intraoperative hypotension that might occur will also be diminished.

## 1. Introduction

Intraoperative hypotension is a frequently occurring event that influences postoperative outcomes and is associated with increased mortality, an increased risk of acute renal failure, and a risk of cardiac complications [1]. During the intraoperative period, even brief episodes of inadequate tissue perfusion due to hypotension can have lasting deleterious effects on organ function and patient survival. In this context, the recognition and management of intraoperative hypotension is critical for optimising patient safety and improving surgical outcomes. Furthermore, an association has been shown between the occurrence of postoperative delirium and intraoperative hypotension [2,3]. This complex neurocognitive disorder has an incidence of up to 20% and is correlated with a substantially increased risk of morbidity and mortality up to one year after surgery [4,5]. Our own data, which include over 30,000 patients over a period of more than 10 years, reinforced these findings and also show that intraoperative hypotension is an independent risk factor for the occurrence of postoperative delirium [6]. The monitoring of haemodynamics is crucial to allow timely intervention during surgery.

The causes of intraoperative hypotension are multifactorial, including a wide spectrum of clinical and iatrogenic factors. Anaesthetic agents, particularly those used to induce general anaesthesia, have well-documented vasodilatory effects and negative inotropic properties that contribute to reductions in systemic vascular resistance and myocardial contractility. These pharmacological actions often precipitate arterial blood pressure declines during induction and maintenance phases of anaesthesia. Moreover, pre-existing comorbidities such as chronic hypertension, heart failure, and autonomic dysfunction significantly modulate the cardiovascular response to anaesthesia and surgical stress. Long-term medications, notably antihypertensive agents such as beta-blockers, ACE inhibitors (angiotensin-converting enzyme inhibitors), and calcium channel blockers, further complicate perioperative haemodynamics by blunting compensatory mechanisms that ordinarily counteract hypotension. The interplay of all these factors requires an individualised, interdisciplinary approach to guarantee haemodynamic stability.

Intraoperative hypotension does not have a universally accepted definition. This complicates efforts to standardise protocols for treatment and research. Since an increased risk of end-organ ischemia and adverse outcomes can be expected when mean arterial pressure falls below 60–70 mmHg, this is generally the threshold at which intraoperative hypotension is defined [7,8]. The precise threshold may vary depending on patient-specific factors, including baseline blood pressure and the presence of cerebrovascular or renal disease. For instance, patients with chronic hypertension may experience ischemic complications at higher MAP (mean arterial pressure) values, emphasising the need for individualised blood pressure targets during surgery.

In the specific case of endovascular aortic valve replacement (TAVI), perioperative haemodynamics are uniquely influenced by procedural factors that transiently alter cardiac loading conditions. Upon implantation of a competent prosthetic valve, there is an acute reduction in cardiac afterload due to the restoration of valve competence and improved forward flow. This haemodynamic shift can precipitate abrupt changes in systemic blood pressure. Additionally, the technique of “rapid pacing,” involving the use of a transvenous pacemaker to temporarily increase heart rate, is often employed during valve deployment. Rapid pacing induces a brief period of severely reduced cardiac output or “low flow” state, which is haemodynamically significant and can precipitate transient hypotension. These unique procedural elements make the intraoperative management of TAVI patients more complex, highlighting the need for sophisticated monitoring and predictive tools.

The Edwards HemoSphere monitor and the Acumen IQ sensor provide an innovative system capable of predictive detection of intraoperative hypotension. The result is a dimensionless metric termed the “Hypotension Prediction Index” (HPI), which ranges from 0 to 100. An HPI value of 85 signifies an 85% likelihood that a hypotensive episode (typically defined as MAP < 65 mmHg) will occur within the next 15 min. This predictive capability is underpinned by a high sensitivity and specificity of 85.8% (95% CI, 85.8–85.9%) for forecasting hypotension five minutes before onset, as reported in validation studies [9,10]. This provides a system with high predictive power that uses values obtained during standard monitoring to provide the anaesthetist with additional information about haemodynamics and end-organ perfusion. It provides the attending anaesthetist with the opportunity to initiate therapeutic measures early to prevent impending hypotension. The algorithm behind HPI, which the manufacturer does not explain in detail [11], does not require additional invasive measures but utilises arterial pressure data already obtained from standard intraoperative monitoring and provides real-time information.

Studies in the field of non-cardiac surgery have shown that the use of HPI alerts leads to significant reductions in the frequency and cumulative duration of intraoperative hypotension [12,13]. Similarly, in cardiac surgery, a patient population characterised by substantial hemodynamic volatility, the HPI algorithm has shown comparable predictive accuracy, providing clinicians with a valuable tool for optimising perioperative haemodynamics [14]. These benefits may translate into improved postoperative outcomes, including decreased incidence of acute kidney injury and shortened hospital stays.

Despite these encouraging data, the applicability and effectiveness of HPI-guided management in the specific context of transfemoral aortic valve implantation remain underexplored. Given the unique haemodynamic challenges posed by TAVI procedures, there is a compelling need to investigate whether HPI monitoring can similarly reduce intraoperative hypotension and improve patient outcomes in this setting.

With this pilot study, we aim to examine whether HPI-guided intraoperative management can reduce the incidence and severity of hypotension during TAVI. We assess the predictive performance of the HPI algorithm in these especially vulnerable patients, and we analyse if the HPI alerts encourage early intervention to possibly diminish episodes of hypotension.

## 2. Materials and Methods

All patients provided written informed consent to participate in this prospective randomised observational study, which was approved by the Ethics Committee of the Ruhr-University, Bochum, Germany (Ethik-Nr. 2022-893). Data from the IQTIG (Institut für Qualitätssicherung und Transparenz im Gesundheitswesen) database were prospectively analysed in this study, which is registered in the German Clinical Trials Register (Deutsches Register Klinischer Studien, DKRS), the national WHO primary registry (DRKS00028749). IQTIG is an independent Institute for Quality Assurance and Transparency in Health Care that evaluates quality contracts between statutory health insurance providers and hospitals as a means to improve inpatient care. These contracts have existed since 2020, introduced by the Federal Joint Committee (G-BA) in Germany.

If complications occurred during the procedure requiring a thoracotomy, the patient was excluded from the study. They underwent a preoperative frailty assessment consisting of various cognitive and functional tests [15] and received perioperative care according to hospital standards. Anaesthetic care was provided by an experienced senior consultant cardiac anaesthetist and a well-trained anaesthetic nurse. This was not always the same team, but all people involved had the same level of training and experience. Intraprocedural monitoring included a 5-lead ECG, continuous end-expiratory capnometry, and oxygen saturation measurements. Arterial pressure measurement was established for extended haemodynamic monitoring. Furthermore, a central venous catheter and a venous sheath for the insertion of a transvenous pacemaker were placed in the internal jugular vein. TAVI was performed under analgesic sedation with propofol (2–3 mg/kg/h) and remifentanil (0.8–1 mg/kg/h), after an initial bolus of 5–10 µg Sufentanil. Administration was weight-based and dosed via syringe pumps. In addition, the depth of anaesthesia was measured using processed EEG monitoring (Narcotrend^®,^ Hannover, Germany) to ensure consistent sedation, and cerebral oxygenation was measured using near-infrared spectroscopy (NIRS). In both study groups, blood pressure was measured invasively with an arterial line, using Edwards Acumen sensors connected to a HemoSphere monitor (Edwards Lifesciences, Irvine, CA, USA). The signal was simultaneously fed to Philips anaesthesia monitors. Vital signs were recorded in the anaesthesia patient data management system (PDMS COPRA, COPRA system GmbH, Berlin, Germany).

Patients were randomly divided into two groups: in the test group, the Edwards monitor with the HPI values was available to the anaesthetist as additional information, whereas in the control group, the HemoSphere monitor was covered and its alarms deactivated. In both groups, all other parameters were available to the attending anaesthetist in the same format as usual.

A mean arterial pressure of 65 mmHg was specified as the lower limit. If this limit was breached, vasopressors and inotropes were administered according to the hospital’s standard operating procedures. The transfemoral aortic valve was implanted according to the cardiological and cardiosurgical standard operating procedures of our institution [16,17], including anticoagulation with unfractioned heparin (150–220 I.U./kgBW), to achieve an activated clotting time above 270 s after femoral catheterization. This was antagonised at the end of the procedure with protamine (100–200 I.U./kgBW). The anticoagulation regime is in line with the regime of previous studies [18,19,20]. Postoperatively, the patient was transferred to the cardiac intensive care unit for post-interventional observation, where HPI monitoring continued until the arterial catheter was removed and the patient was transferred to the general ward.

The primary endpoint of the study is the time-weighted average of intraoperative hypotension, which is calculated from the intensity and duration of hypotension, adjusted for the duration of surgery (TWA_65_), using the following formula:

$$\frac{\sum_{i=0}^{n}(d_{t_{i}}-d_{t_{i-1}})\cdot {MAP}_{t_{i}}}{\mathrm{Duration}\;\mathrm{of}\;\mathrm{Surgery}}\forall i:{MAP}_{t_{i}}<65[mmHg]$$

The sum over all time points where the MAP is <65 mmHg is multiplied by the mean arterial pressure at time point *t_i_*. This is divided by the total duration of surgery.

Secondary endpoints are the cumulative time of hypotensive episodes in minutes and—again—adjusted for the duration of the procedure (TWA_total_).

Documentation was recorded in a pseudonymized, digital form on computers available at the institution.

Statistical analysis was performed with Python 3.11.4 [21], R version 4.5.0 [22] and ggplot2 version 3.5.2 [23], and Excel for Mac version 16.51. Python was used to programmatically retrieve intraoperative vital signs and therapy data. The resulting data were manually checked for plausibility. Summary statistics were calculated with base R and graphics, and statistical tests were calculated using ggplot2. Excel was used for multivariate analysis. Between-group differences were calculated with the Mann–Whitney U test due to non-normal distributions. A significance level of *p* < 0.05 was applied for all statistical tests.

## 3. Results

A total of 50 patients undergoing transfemoral aortic valve implantation from March 2022 to July 2022 were included in this study, 22 of them were randomised to the control group and 28 to the intervention group. Demographical data are presented in Table 1.

**Figure 1:**
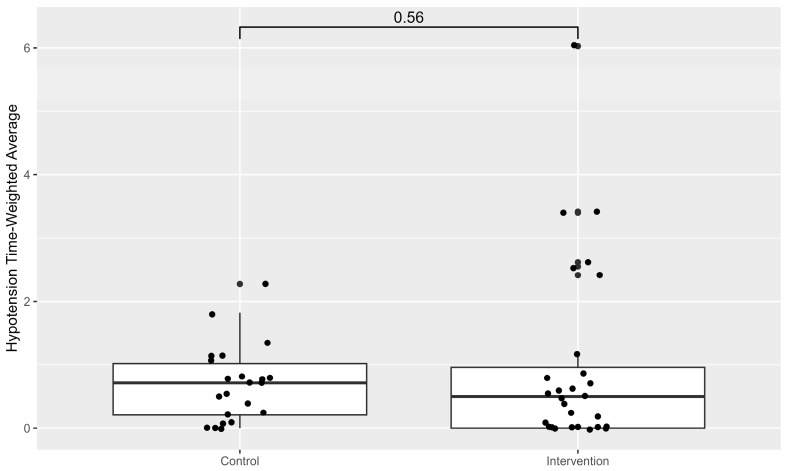
Box-plot of TWA_65_ in both groups.

No differences in the TWA_65_, which is the primary endpoint, could be detected between both groups (Figure 1).

**Figure 2:**
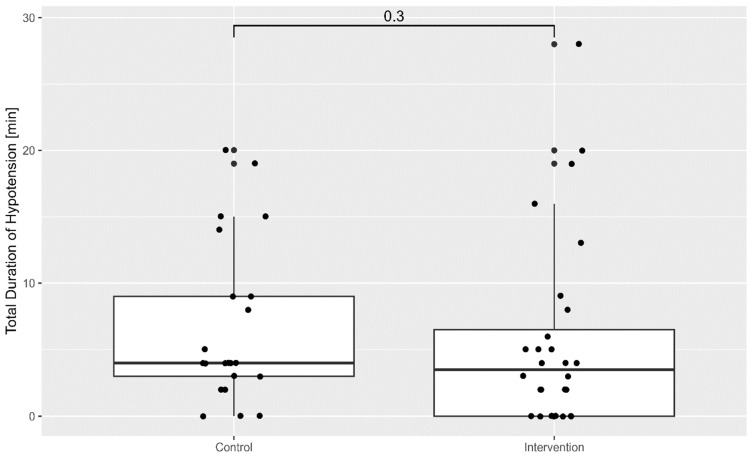
Box-plot of the total duration of hypotension in both groups.

The cumulative duration of hypotension throughout the procedure was lower in the intervention group, as shown in Figure 2.

**Figure 3:**
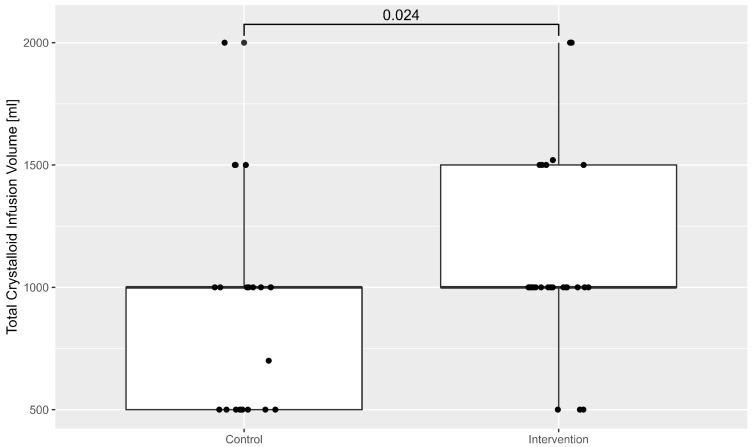
Box-plot of the total volume administered during TAVI in both groups.

The administration of intravenous volume was also higher in the intervention group. This is illustrated in Figure 3.

The influence of preoperative creatinine and haemoglobin on TWA_65_, as tested in a linear regression model, showed no significant correlation (R^2^ = 0.06; F (2, 47) = 1.5; *p* = 0.24).

## 4. Discussion

The results of the present prospective observational pilot study demonstrate several important findings regarding the management of intraoperative hypotension during TAVI.

(1). No difference in intraoperative hypotension in terms of TWA_65_ between control and intervention group could be detected. Still, (2). the overall duration of intraoperative hypotension was reduced in the intervention group and (3). the administration of intraoperative volume was higher in the intervention group when compared to controls.

In this pilot study it could be demonstrated that managing haemodynamics during transcatheter intervention with the help of the HPI is not inferior to relying on arterial blood pressure measurement alone. Our findings are in line with previous studies [24], which showed that guidance with HPI during anaesthetic care in non-cardiac surgery did not significantly reduce the incidence of hypotension when compared to conventional monitoring. HPI guidance can provide important predictive data but does not seem to be suitable for universally preventing all episodes of hypotension.

Possible reasons for this might be the nature of the TAVI procedure itself. Patients’ haemodynamics during TAVI can be suddenly affected due to mechanical manipulation of the ventricle and/or the aorta. Also, blood pressure can drop due to the rapid ventricular pacing employed during the deployment of the new valve [25,26,27]. These events typically cause abrupt drops in blood pressure that occur too swiftly to be anticipated by predictive software such as HPI. Consequently, the anaesthetist may not have the opportunity or indication to intervene with fluid administration or vasopressors during these transient events, as these hypotensive episodes generally resolve immediately after the cessation of rapid pacing or manipulation. This means that certain periods of hypotension are expected and unavoidable, so predictive monitoring systems will provide only limited predictive power during these intervals.

Despite these challenges, a key finding of our study is that the cumulative time spent in hypotension throughout the procedure was reduced in patients monitored with HPI. When analysing the total time in which the mean arterial pressure (MAP) remained below 65 mmHg, the early warning provided by the HPI alarm facilitates more effective prevention of hypotensive episodes overall. This is likely because the HPI system offers not only a prediction of impending hypotension but also detailed haemodynamic parameters including stroke volume variation (SVV), dP/dt (rate of pressure change in the ventricle throughout time), cardiac output, and cardiac index [9]. These additional metrics provide the anaesthetist with insights into the underlying causes of hypotension, whether related to preload, contractility, or afterload, enabling timely and targeted interventions.

The finding that the intervention group received more intravenous volume intraoperatively supports this interpretation. The increased volume administration could reflect the anaesthetist’s response to early HPI warnings, proactively addressing vasodilation or hypovolemia before hypotension becomes clinically significant. This is important as patients undergoing TAVI often experience vasodilation secondary to analgosedation and may be volume-depleted due to preoperative fasting protocols [28]. Early fluid administration based on haemodynamic monitoring thus could contribute to improved stability in blood pressure during the procedure.

The clinical implications of the findings of our pilot study are crucial. Maintaining haemodynamic stability during TAVI is critical due to the vulnerability of this patient population, who are often elderly with multiple comorbidities [29]. Episodes of intraoperative hypotension have been linked to adverse outcomes such as acute kidney injury, myocardial injury, and increased mortality in various surgical populations [30,31]. Therefore, any intervention that reduces the cumulative burden of hypotension can improve postoperative outcomes. Our results suggest that HPI-guided monitoring might enhance the anaesthetist’s ability to maintain more consistent blood pressure control during TAVI, which could possibly translate into fewer postoperative complications.

Future research should focus on confirming these preliminary findings in larger, multicentre randomised controlled trials. Such studies would better delineate the efficacy of HPI-guided haemodynamic management compared to standard monitoring and explore its impact on clinical outcomes, including postoperative morbidity and mortality. Furthermore, it remains to be determined whether the benefits observed are primarily attributable to the HPI’s predictive alert system or to the additional detailed monitoring of cardiac output determinants—preload, afterload, and contractility.

Moreover, the development of algorithms that can better anticipate abrupt haemodynamic changes specific to TAVI, such as those related to rapid pacing and valve manipulation, might further improve patient outcomes. Integrating data from additional sources like electrocardiographic changes, procedural milestones, or real-time imaging might provide a more comprehensive picture for predictive monitoring systems. Such advancements could transform haemodynamic management during complex cardiovascular interventions.

Intraoperative hypotension remains a critical challenge in perioperative medicine, with far-reaching implications for patient morbidity and mortality. Advances in machine learning and real-time monitoring technologies, exemplified by the HemoSphere and HPI system, offer promising avenues for proactive management. Through the targeted prediction and prevention of hypotensive episodes, these innovations might be able to transform intraoperative care, particularly in high-risk procedures such as TAVI. Continued research is essential to validate these tools across diverse surgical populations and to establish evidence-based protocols that optimise their use.

### Limitations

A limitation of our study is that it included patients from only a single institution. This may limit the generalisability of the results to a broader patient population or different procedural environments. However, the advantage of this single-centre approach is that it allowed for a highly standardised assessment of the HPI system under consistent procedural protocols and patient characteristics. To our knowledge, the use of HPI monitoring during TAVI has not yet been described elsewhere, making this study a valuable initial exploration into the application of this technology in a novel clinical context.

Our cohort consists of 50 patients, which might lead to low statistical power to detect significant results and to an increased risk of type 2 error. Still, being a pilot study, it first describes the use of advanced haemodynamic monitoring during TAVI and opens the way for future research to confirm our findings. Notably, the influence of other confounders, such as co-morbidities and premedication, must be assessed.

It was demonstrated in the past [32] that female sex is an independent risk factor for access-site bleeding after percutaneous intervention. Whether this is true for the development of hypotension during the procedure has not yet been analysed. In this present pilot study, the distribution of sex in both groups was rather heterogenous, and this should be ruled out in future research. The same is true for other confounders, like the co-morbidity burden.

## 5. Conclusions

Data from the present pilot study suggest that HPI monitoring could be a tool to stabilise haemodynamics. We conclude that HPI monitoring during TAVI could be a feasible strategy to reduce the overall duration of intraoperative hypotension, thus improving haemodynamic stability. Although not all hypotensive episodes are prevented, particularly those related to procedural manipulations, the early warning system might enable the caring anaesthetist to intervene effectively. The same is true for the detailed haemodynamic insights that are provided by the system.

As the stability of the mean arterial blood pressure is crucial in these vulnerable and fragile patients, the integration of HPI during TAVI into the anaesthetic regime should be assessed in future research.

## Figures and Tables

**Table 1:** Demographical data of the study cohort. Age, BMI and the duration of the procedure and anaesthesia are presented as mean [minimum; maximum], all other data as the total amount (percentage of the group). BMI: body mass index; atr. fib.: atrial fibrillation; renal insuf.: renal insufficiency; art. HT: arterial hypertension; LV: left ventricular function; diab. mell.: diabetes mellitus; cer. vasc. dis.: cerebral vascular disease; COPD: chronic obstructive pulmonary disease; CAD: coronary artery disease; prev. MI: previous myocardial infarction; self-exp.: self-expanding valve; balloon-exp.: balloon-expanding valve; dur. proc.: duration of the procedure in minutes; dur. anaesth.: duration of anaesthesia in minutes.

	Overall	Control	Intervention
Variable	50	22	28
Sex (male)	26 (52%)	11 (39%)	15 (68%)
Age (years)	83 [67; 95]	83 [67; 92]	82 [69; 95]
BMI	27.6 [19.7; 43.6]	28.2 [19.7; 43.6]	27.2 [20.1; 35.2]
Sinus rhythm	33 (66%)	13 (59%)	20 (71%)
Atrial fib.	12 (24%)	5 (23%)	7 (25%)
Renal insuf.	12 (24%)	6 (27%)	6 (21%)
Art. HT	37 (74%)	15 (68%)	22 (78%)
LV ≥ 50%	39 (78%)	18 (82%)	21 (75%)
LV 40–49%	1 (2%)	0	1 (3%)
LV 30–39%	6 (12%)	3 (14%)	3 (11%)
LV < 30%	4 (8%)	1 (4%)	3 (11%)
Diab. mell.	14 (28%)	4 (18%)	10 (36%)
Cer. vasc. dis.	8 (16%)	1 (4%)	7 (25%)
COPD	4 (8%)	2 (9%)	2 (7%)
CAD	34 (68%)	15 (68%)	19 (68%)
Prev. MI	8 (16%)	2 (9%)	6 (21%)
Self-exp.	22 (44%)	4 (18%)	18 (64%)
Balloon-exp.	28 (56%)	18 (82%)	10 (36%)
Dur. proc.	60 [29; 189]	65 [37; 189]	50 [29; 75]
Dur. anaesth.	160 [109; 267]	168 [124; 267]	149 [109; 198]

## Data Availability

Due to local legislation and privacy regulations, data can be made available only after signing a collaboration agreement.

## Abbreviations

The following abbreviations are used in this manuscript:

TAVI	Transfemoral aortic valve implantation
HPI	Hypotension prediction index
TWA	Time-weighted average
ECG	Electrocardiography
EEG	Electroencephalography
NIRS	Near-infrared spectroscopy
